# Sex composition and its impact on future childbearing: a longitudinal study from urban Uttar Pradesh

**DOI:** 10.1186/s12978-018-0482-y

**Published:** 2018-02-27

**Authors:** Sowmya Rajan, Priya Nanda, Lisa M. Calhoun, Ilene S. Speizer

**Affiliations:** 1 Global Health Innovations Center, Duke University, Durham, NC 27701 USA; 2 Bill & Melinda Gates Foundation, New Delhi, India; 3 Carolina Population Center, University of North Carolina at Chapel Hill, Chapel Hill, USA; 4 Gillings School of Global Public Health, University of North Carolina at Chapel Hill, Chapel Hill, USA

**Keywords:** Son preference, Sex composition, India, Parity progression, Contraceptive use, Fertility desires

## Abstract

**Background:**

The sex composition of existing children has been shown to influence childbearing decision-making and behaviors of women and couples. One aspect of this influence is the preference for sons. In India, where son preference is deeply entrenched, research has normally focused on rural areas using cross-sectional data. However, urban areas in India are rapidly changing, with profound implications for childbearing patterns. Yet, evidence on the effect of the sex composition of current children on subsequent childbearing intentions and behavior in urban areas is scant. In this study, we analyze the impact of sex composition of children on subsequent (1) parity progression, (2) contraceptive use, and (3) desire for another child.

**Methods:**

We analyze prospective data from women over a four year period in urban Uttar Pradesh using discrete-time event history logistic regression models to analyze parity progression from the first to second parity, second to third parity, and third to fourth parity. We also use logistic regression models to analyze contraceptive use and desire for another child.

**Results:**

Relative to women with no daughters, women with no sons had significantly higher odds of progressing to the next birth (parity 1 – aOR: 1.31; CI: 1.04–1.66; parity 2 – aOR: 4.65; CI: 3.11–6.93; parity 3 – aOR:3.45; CI: 1.83–6.52), as well as reduced odds of using contraception (parity 2 – aOR:.58; CI: .44–.76; parity 3 – aOR: .58; CI: .35–.98). Relative to women with two or more sons, women with two or more daughters had significantly higher odds of wanting to have another child (parity 1 – aOR: 1.33; CI: 1.06–1.67; parity 2 – aOR: 3.96; CI: 2.45–6.41; parity 3–4.89; CI: 2.22–10.77).

**Conclusions:**

Our study demonstrates the pervasiveness of son preference in urban areas of Uttar Pradesh. We discuss these findings for future programmatic strategies to mitigate son preference in urban settings.

## Plain English summary

In India, son preference is prevalent, and has been shown to influence parental reproductive decision making and behavior. When parents have a strong son preference but do not have their desired number of sons, they are more likely to want to continue childbearing, less likely to use contraception, and more likely to have more children. In this study, we use longitudinal data from urban Uttar Pradesh to examine how the sex composition of current children influences future reproductive intentions and behavior. Specifically, we examine if the sex composition of children is associated with parity progression, modern contraceptive use, and desire for an additional child.

We use baseline and endline data from the Measurement, Learning, & Evaluation Project in urban Uttar Pradesh. Our findings show that at parities one, two, and three, women continue to have children if they have no son or only one son at baseline. Having no sons (or only daughters) at baseline is also associated with lower odds of using modern contraception, and higher odds of wanting another child at endline.

This study shows that the sex composition of children is associated with women’s future reproductive decision-making and behavior, including greater desire for additional children, more births, and less modern contraceptive use. Interventions to mitigate the negative effects of son preference must emphasize both the intrinsic value of girls, and the deleterious consequences of high-parity births and low contraceptive use.

## Background

A strong preference for sons over daughters has been documented in India for much of the last century [1–5]. Termed “son preference,” this phenomenon is also prevalent in several countries in South and East Asia, such as Bangladesh, Nepal, China, and Vietnam [1, 4]. Parents value sons over daughters for various social, economic, and religious reasons [2, 3, 6]. For instance, daughters are considered a burden because of large dowry and marriage expenses, their exclusion and discrimination in inheritance laws, and lower value as agricultural labor [6]. These and other reasons have normalized a strong preference for sons in several regions of India, and consequent discrimination against daughters in childhood health, nutrition, and mortality [1]. However, even in countries with a strong preference for sons, many parents also want to have at least one daughter [6]. This has been true in India as well, where Hindu parents earn merit for giving away a daughter in marriage without expecting anything in return (*kanya daan*) [6].

Parents pursue different strategies to implement their preference for sons over daughters [5]. Over the last three decades, a commonly used strategy to achieve the desired number/ proportion of sons has been sex-selective abortion [5]. The widespread availability and use of sex selection technologies (such as ultrasound and amniocentesis) in India and East Asian countries such as South Korea and Taiwan has contributed to severely distorted sex ratios at birth (over 1.10 boys to girls) [5, 7, 8].

Another strategy that parents employ is adopting different stopping or contraception rules for childbearing depending on the sex composition of their existing children [5, 9]. In other words, parents continue having children until they achieve their desired number (or proportion) of sons [5, 6]. Continuing to bear children until parents reach their desired number of sons would not only increase the number of unwanted daughters within families, but also unwanted and total fertility in the aggregate [3]. In India, studies have shown that couples who do not have their desired number of sons are less likely to use contraception, more likely to have shorter birth intervals, more likely to want additional children, and more likely to progress to higher parities [6, 10–14].

The current approach to studying son preference in families is by examining the association between the sex composition of children and various outcomes by using cross-sectional data; many of these studies focus on rural areas or national samples [1, 3, 6, 9, 14–16]. Our study contributes to existing literature by using longitudinal data from urban areas of Uttar Pradesh. Specifically, we hypothesize that the sex composition of current children influences subsequent childbearing intentions and behaviors over a four year study period in urban Uttar Pradesh. We approached our analyses with the following hypotheses among women at the first, second, and third parities:

1. Parity progression (to second, third, and fourth parities) will be higher among women with a daughter-dominant sex composition of current children.
2. Modern contraceptive use will be lower among women whose children have a daughter-dominant sex composition.
3. Desire for additional children will be greater among women whose children have a daughter-dominant sex composition.

## Study context

Uttar Pradesh, situated in the North of India, is the most populous state with nearly 200 million individuals, according to the 2011 Census [17]. The state has a poor record in health and human development and suffers from weak public health infrastructure, in regards to quality of service delivery and capacity of trained providers [18]. It also ranks high in infant and child mortality, and low in modern contraceptive use and women’s education [19]. Further, Uttar Pradesh has consistently had among the highest total fertility rates (TFR) in the country [20]: while TFR has been declining in India for the previous two decades with about 2.6 children per woman in 2008, it was 3.8 children per woman in Uttar Pradesh in the same year [21]. However, data from the 2015 to 16 National Family Health Surveys show that TFR is now steadily declining in Uttar Pradesh as well, with 2.7 children per woman in the state, and 2.1 children per woman in its urban areas [19].

## Methods

In 2009, the Bill & Melinda Gates Foundation launched the Urban Reproductive Health Initiative (URHI) in select cities in four countries – India, Kenya, Nigeria and Senegal –with a particular focus on the urban poor. The goal of the program was to improve access to and use of family planning and reproductive health services in urban areas. At the same time, the Measurement, Learning & Evaluation (MLE) Project, led by the Carolina Population Center at the University of North Carolina at Chapel Hill, was funded to perform rigorous impact evaluation of the URHI programs in all four countries. In India, the URHI project was called the Urban Health Initiative (UHI), and fielded in urban sites of the state of Uttar Pradesh (UP) in late 2010. The program was deployed and evaluated in six cities– Agra, Aligarh, and Moradabad from western UP, and Allahabad, Gorakhpur, and Varanasi from eastern UP [22]. These cities were selected by UHI in conjunction with the Government of Uttar Pradesh and the Government of India, based on criteria such as their population size, geographic and regional diversity, large slum populations, and low contraceptive prevalence [13, 22]. Estimates from the provisional population totals from the 2011 Census show that these six cities contributed to 18% (or 8.0 million people) of the total 44.5 million urban residents in the state [17].

The MLE Project undertook longitudinal surveys across three periods to collect baseline, midterm, and endline data. MLE used a multi-stage sampling design to collect baseline data in 2010 from a representative sample of households and women in each city. An important component of the sampling strategy was the oversampling of slums in each city to evaluate and examine program activities targeted towards the urban poor [22]. Prior to selection of primary sampling units, all cities were mapped to identify the location and boundaries of registered slums from the government’s list. In a second step, to identify additional slums, densely populated areas with poor access to water and sanitation services were mapped during the process of ground truthing^1^ the registered slums [23, 24]. In each city, half of selected primary sampling units were slum areas and the other half were non-slum areas; this permitted obtaining an over sample of the urban poor. In each selected primary sampling unit, a random sample of 30 households was selected for a detailed household interview with the head of the household as well as interviews with all currently married women ages 15–49 years. Among the topics covered in the women’s survey were basic sociodemographic characteristics, reproductive preferences and behavior, maternal and child health services, contraceptive knowledge and use, media use and gender relations. In 2014, the endline survey was conducted in which field teams sought to find all women who were usual residents at baseline, including those that were no longer married and were outside the age range of 15–49 years, and still residing in a study city in order to measure program exposure and changes in contraceptive use and fertility behaviors. The response rate for the endline survey was 83.6% [24]. In order to make the sample truly representative of the population, we use endline weights in the descriptive analyses reported in this study. All MLE surveys and study procedures were approved by the Institutional Review Board Committees of the University of North Carolina at Chapel Hill, ICRW and Mamta-Health Institute for Mother and Child in India.

A total of 14,043 women had complete interviews at baseline and endline. While nonresponse and attrition from baseline to endline could be a source of bias, with a response rate of 83.6%, we believe that this bias will not affect our study findings substantially [15]. Additionally, by weighting all analyses, we hope to also adjust for nonresponse bias. For the analyses described in this study, we focus on the sample of women who had at least one birth, but not more than three births at baseline, were not sterilized, menopausal, infecund or pregnant at baseline, and were re-interviewed at endline. The focus on this sub-sample relates to our interest in how sex composition of previous children influence parity progression, contraceptive use, and subsequent fertility intentions which means we need to restrict our sample to women who had at least one birth. Further, because over 75% of births at baseline were in parities 1 to 3, we also restrict our analyses to these critical parities. Last, because fertility intentions are recorded only for women who were fecund (not sterilized and menopausal), and not pregnant at the time of baseline interview, we include the sample of women who had valid reports of fertility intentions at baseline.

We analyzed three outcome variables for this study. The first outcome of interest is whether each respondent progressed from parities one, two, and three to parities two, three, and four respectively between baseline and endline. These variables are coded as binary variables for each parity transition. The second outcome relates to contraceptive use to examine if respondents used modern contraception at endline (use of modern method vs. no or traditional method). The last outcome examines desire for another child at endline: want another child (soon or later) vs. want no more children.

The key predictor variable is the sex composition of living children at parities one, two, and three at the time of the baseline interview. At parity one, the two possible categories for sex composition of existing children are: (1) one son (reference) and (2) one daughter. At parity two, the categories of sex composition of existing children are: (1) two sons (reference), (2) one son and one daughter, and (3) two daughters. At parity three, the categories of sex composition of children are: (1) three sons(reference), (2) two sons, one daughter, (3) two daughters, one son, and (4) three daughters. We chose the reference categories to indicate the son-dominant category within each parity, where applicable. The son-dominant category at parity one is one son; at parity two, it is two sons; and at parity three, it is three sons.

For all three models, controls include standard demographic variables that affect the outcomes. These include age at baseline (15–24 years, 25–34 years, and over 35 years), education (no education, primary, secondary, and more than secondary), religion (Hindu, and other religion), household wealth quintile (poorest, poor, medium, rich, and richest), city (Agra, Aligarh, Allahabad, Gorakhpur, Moradabad, and Varanasi), and slum residence (slum, non-slum). Household wealth quintiles were constructed similar to the Demographic and Health Surveys and as described by Filmer and Pritchett [25]. To elaborate, wealth indices were constructed using data on the ownership of consumer goods, assets, and materials used to construct the house/ dwelling, at the household level, comprising 27 different variables. This information was used to conduct principal components analysis and estimate a factor (or wealth) score for each household. Households were then placed in quintiles, ranging from lowest (poorest) to highest (richest). We also include baseline fertility intentions for another child (want another child, and want no more children), as well as any contraceptive use at baseline (yes/ no).

Because all our outcome variables are binary, we use logistic regressions for all multivariate analyses. First, for parity progression, we use a series of discrete-time logistic regressions predicting whether the respondent had a second, third, or fourth birth since the time of their first, second, or third birth respectively. Second, we use logistic regression to estimate modern method use at endline (modern method vs. no/ traditional method). Next, we use logistic regressions to predict endline fertility intentions (want another child vs. want no more). For all outcomes, we show the adjusted estimates controlling for baseline sociodemographic characteristics, fertility intentions and FP use. We present results from the logistic regression models as odds ratios, which can be interpreted as follows: if the odds ratio is greater than 1.0, the association of the particular independent variable with the outcome is positive; if the odds ratio is less than 1.0, the association of the particular independent variable with the outcome is negative. All models control for clustering in the data using the svy commands in Stata statistical software.

## Results

### Sociodemographic characteristics

Table 1 presents key descriptive statistics showing the characteristics of mothers with one, two or three children at baseline who were not pregnant or infecund (menopausal, hysterectomy, sterilized) at the time of the baseline interview, and followed up at endline four years later (*N* = 5761). More than half of the women in our sample were between 25 and 34 years old at baseline (51.73%); and more than a quarter of them were 35 years and older (27.63%). Nearly a fifth of the women in this sample had no education (19.21%), a third had more than secondary school education (33.66%), and nearly 40% had completed secondary school education (39.41%). The majority of this sample was Hindu (80.39%), and resided in non-slums (84.10%). Over half of the sample was from households in the two highest quintiles – rich and richest (55.15%).

**Table 1:** Baseline characteristics of married, non-pregnant, fecund mothers with one, two or three children at baseline (and followed up endline)

Characteristic	Percent	*N* = 5761
Age (years)		
15–24	20.65	1189
25–34	51.73	2980
Over 35 years	27.63	1592
Education		
None	19.21	1106
Primary	7.73	446
Secondary	39.41	2270
More than secondary	33.66	1939
Religion		
Hindu	80.39	4630
Other	19.61	1131
Wealth quintile		
Poorest	11.17	643
Poor	14.25	821
Medium	19.43	1120
Rich	25.97	1496
Richest	29.18	1681
Slum residence		
Slum	15.90	915
Non slum	84.10	4846
City		
Agra	23.10	1331
Aligarh	12.78	736
Allahabad	20.48	1180
Gorakhpur	15.79	910
Moradabad	9.70	558
Varanasi	18.15	1046
Number of children		
1	30.80	1775
2	44.41	2558
3	24.79	1428
Number of sons		
0	22.96	1323
1	52.18	3006
2	21.80	1256
3	3.06	176
Number of daughters		
0	32.22	1857
1	48.40	2789
2	17.50	1009
3	1.87	108
Fertility intentions		
Want soon/ want later	33.09	1906
Want no more	66.91	3855
Contraceptive use		
No method	27.01	1556
Modern method	47.63	2744
Traditional method	25.36	1461

Note: Endline weights used for percentages and number of observations

In this study sample, over 40% had two children (44.41%), nearly a third had one child (30.80%), and a quarter had three children (24.79%). Among these women, just over a fifth had no sons (22.96%), whereas nearly a third had no daughter (32.22%). More than half of the sample had one son (52.18%), whereas a little under half of the sample had one daughter (48.40%). Nearly two thirds of the women wanted no more children at baseline (66.91%), but less than half were using a modern contraceptive method (47.63%) at baseline.

### Sex composition and parity progression

Table 2 shows the results of the multivariate logistic regression analyses of the sex of previous child(ren) on women’s parity progression from the first, second, and third births between baseline and endline. Although not presented in the multivariate tables, we controlled for baseline sociodemographic characteristics, fertility intentions, and contraceptive use in all models. Our findings show patterns of association between the sex composition of previous children and subsequent childbearing that are consistent with a preference for sons. Model 1 shows that in the transition from first to second birth, women with one daughter had significantly higher odds of progressing to a second birth (aOR: 1.31; 95% CI: 1.04–1.66) compared to women with one son. Model 2 shows the association between sex of previous children in the transition to the third birth. The adjusted coefficients show that relative to women whose two children were both sons, those who had one son and one daughter had increased odds of progressing to a third birth (aOR: 1.65; 95% CI: 1.21–2.26). Women whose two children were both daughters had more than four times higher odds of progressing to a third birth (aOR: 4.65; 95% CI: 3.11–6.93). Model 3 shows the association of sex composition in the transition to the fourth birth. Relative to women with three sons, those with three daughters had over three times higher odds of progressing to a fourth birth (aOR: 3.45; 95% CI: 1.83–6.52).

**Table 2:** Odds Ratios from Logit Regression of Baseline Sex Composition on Having Second, Third, and Fourth Child After Baseline

Sex composition of previous children at baseline	Odds ratios (95%CI)	*P*
Model 1		
*Parity 1 (N = 1661)*	*(parity 1 - > parity 2)*	
1 son (ref)	1.00	–
0 sons, 1 daughter	1.31	.022
	(1.04–1.66)	
Model 2		
*Parity 2 (N = 2490)*	*(parity 2 - > parity 3)*	
2 sons (ref)	1.00	–
1 son, 1 daughter	1.65	.002
	(1.21–2.26)	
0 sons, 2 daughters	4.65	.000
	(3.11–6.93)	
Model 3		
*Parity 3 (N = 1610)*	*(parity 3 - > parity 4)*	
3 sons (ref)	1.00	–
2 sons, 1 daughter	.74	.269
	(.44–1.25)	
1 son, 2 daughters	1.51	.100
	(.92–2.48)	
0 sons, 3 daughters	3.45	.000
	(1.83–6.52)	

*Note*: All models adjust for respondent’s baseline age, education, religion, household wealth, slum and city residence, fertility intentions, and contraceptive use

### Sociodemographic variables, fertility intentions, and parity progression (not shown)

Fertility intentions reported at baseline were also strong predictors of subsequent childbearing for the progression to second and third births. Relative to women who want another child, those who want no more children had significantly lower odds of progressing to the next birth. Among the other variables, age has a curvilinear relationship with childbearing: younger women in the prime reproductive years have greater odds of progressing to the next parity, whereas the oldest women have reduced odds of transitioning to the next parity. Women from wealthier households have lower odds of progressing to a subsequent birth. Education, religion, city of residence, and slum residence were not consistently associated with progressing to the next birth.

### Sex composition and modern contraceptive use

In Table 3, we present odds ratios and 95% confidence intervals of the association between sex composition at baseline and modern method use at endline by parity, net of baseline sociodemographic characteristics and fertility intentions. Model 4 shows that among women with one child at baseline, there was no significant association between the sex of that child and the odds of using modern contraception at endline. Model 5 provides evidence that among women with two children, modern method use was associated with the sex of previous children. Specifically, at parity two, relative to women who had two sons, women who had one son and one daughter at baseline had lower odds of using modern contraception at endline (aOR: 0.82; 95% CI: .67–.99). Women who had two daughters relative to two sons had significantly lower odds of using modern contraception (aOR: 0.58; 95% CI: .44–.76). Model 6 shows that women who had three daughters at baseline had significantly lower odds of using modern contraception, relative to women with three sons (aOR: 0.58; 95% CI: .35–.98).

**Table 3:** Odds Ratios from Logit Regression of Baseline Sex Composition on Modern Contraceptive Use at Endline

Sex composition of previous children at baseline	Odds ratios (95%CI)	*P*
Model 4		
*Parity 1 (N = 1661)*		
1 son (ref)	1.00	–
0 sons, 1 daughter	1.04	.723
	(.84–1.29)	
Model 5		
*Parity 2 (N = 2490)*		
2 sons (ref)	1.00	–
1 son, 1 daughter	.82	.045
	(.67–.99)	
0 sons, 2 daughters	.58	.000
	(.44–.76)	
Model 6		
*Parity 3 (N = 1610)*		
3 sons (ref)	1.00	–
2 sons, 1 daughter	.83	.314
	(.58–1.19)	
1 son, 2 daughters	.76	.144
	(.53–1.09)	
0 sons, 3 daughters	.58	.043
	(.35–.98)	

*Note*: All models adjust for respondent’s baseline age, education, religion, household wealth, slum and city residence, fertility intentions, and contraceptive use

### Sociodemographic variables, fertility intentions and contraceptive use (not shown)

Women who did not want more children at baseline had substantially increased odds of using modern contraception in every parity. At every parity, younger women had higher odds of using modern contraception. Women who had completed at least secondary schooling were significantly more likely to use modern contraception at all parities. Wealth and religion were not significantly associated with modern method use across parity.

### Sex composition and desire for another child

Table 4 presents the results of the association between sex composition of children at baseline and desire for an additional child at endline, net of sociodemographic characteristics and fertility intentions. Model 7 in Table 4 shows that the association between baseline sex composition and desire for another child at endline is positive and significant among women with one child at baseline. Specifically, at parity one, women who had one daughter rather than one son at baseline had significantly higher odds of wanting another child at the endline interview (aOR: 1.33; 95% CI: 1.06–1.67). In Model 8, relative to women with two sons, those with two daughters had nearly four times the odds of wanting another child at endline, among women with two children (aOR: 3.96; 95% CI: 2.45–6.41). At parity 3, we combine the categories of women who had three sons, and those with two sons and a daughter, because of the very small cell size of women with three sons who wanted another child (*n* = 5). Similarly, we combine the categories of women who had three daughters and those with two daughters and a son because of the relatively small cell size of women with three daughters and who wanted another child (*n* = 42). Relative to women with two or more sons, those with two or more daughters at baseline had nearly five times the odds of wanting another child (aOR: 4.89; 95% CI: 2.22–10.77).

**Table 4:** Odds ratios from Logit Regression of Baseline Sex Composition on Desire for another Child at Endline

Sex composition of previous children at baseline	Odds ratios (95%CI)	*P*
Model 7		
*Parity 1 (N = 1661)*		
1 son (ref)	1.00	–
1 daughter	1.33	.013
	(1.06–1.67)	
Model 8		
*Parity 2 (2490)*		
2 sons (ref)	1.00	–
1 son, 1 daughter	.68	.082
	(.44–1.05)	
0 sons, 2 daughters	3.96	.000
	(2.45–6.41)	
Model 9		
*Parity 3 (1610)*		
2 or more sons (ref)	1.00	–
2 or more daughters	4.89	.000
	(2.22–10.77)	

*Note*: All models adjust for respondent’s baseline age, education, religion, household wealth, slum and city residence, fertility intentions, and contraceptive use

### Sociodemographic variables, fertility intentions and desire for another child (not shown)

Baseline desire for another child is a strong predictor of desire for another child at endline: women who did not want another child at baseline had significantly lower odds of wanting another child at endline. Desire for another child declined with age in the third parity: it was highest at young ages and gradually declined as women age. Neither wealth nor education was associated with the desire for another child at endline. Women who were not Hindu were less likely to want another child at endline.

## Discussion

Urban areas of Uttar Pradesh, a state in Northern India, provide an opportune site to examine the evolution of reproductive behavior in the context of strong son preference. Our study shows that the sex composition of a woman’s child(ren) is related to her subsequent fertility trajectory. Using prospective data, we found that the sex of a woman’s previous children strongly influences whether she desires another child, will use contraception, and has a subsequent birth.

We highlight three main findings from our study. First, in line with our first hypothesis, women in this urban Uttar Pradesh sample prefer to have a sex composition for their children that is son-dominant, and this desire is manifested in their childbearing behavior. Our findings suggest that at every parity, women who have a sex composition that is daughter-dominant are more likely to progress to the next parity. These associations are substantial and significant, and suggest that women who do not have a son-dominant sex composition are more likely to continue childbearing.

Second, we find modest and inconsistent support for our second hypothesis. Women with a daughter-dominant sex composition base their decision to use modern contraception only after the first parity. In the first parity, the sex of the child has no association with women’s use of modern contraception, suggesting a desire for two children. At parity two, both women with no sons and women with one son are less likely to use modern contraception, relative to those with two sons (son-dominant sex composition). Parity three presents a different picture: we find that women with one, two, or three sons are not significantly different from each other in their use of modern contraception. It is only when women in the third parity have no sons (daughter-dominant sex composition) that they are significantly less likely to use modern contraception.

Last, consistent with our third hypothesis, we find that women with a daughter-dominant sex composition of their children at baseline are more likely to want to have another child at endline. This association is evident in all three parities.

Our findings are consistent with earlier studies from India that show an association between sex composition of previous children and reproductive outcomes such as parity progression, desire for an additional child, and contraceptive use [3, 6, 9, 10, 13, 26]. In general, using cross-sectional data, these studies show that women with fewer sons than daughters (or daughter-dominant sex composition) are more likely to desire another child, more likely to continue to have children, and less likely to use effective contraception [3, 6, 9, 10, 13, 26]. For instance, using baseline MLE data, Calhoun et al. [13] find that son-dominant sex composition is associated with desire for another child: women with more sons than daughters are more likely to want no more children, relative to those with more daughters than sons. Other studies show that regardless of the number of daughters, couples have at least one or two sons before they begin to use contraception [26]. In the same vein, Jayaraman et al. [3] show that the desire for an additional child decreases with the number of sons in the family. In contrast, some studies note a preference for at least one daughter [6, 13, 27]– a finding that is not evident in the parity progression models of this study.

Our findings extend the breadth of earlier studies by examining these outcomes over a four year follow-up period, and by accounting for women’s baseline fertility intentions, contraceptive use, and a variety of sociodemographic controls in predicting future childbearing intentions behavior. Almost all the studies that examine son preference use cross-sectional surveys. Although cross-sectional studies serve many useful purposes, a main drawback is that they do not allow us a sequential understanding of women’s reproductive behavior. However, reproductive decision-making follows an inherently sequential pattern [26, 28], and childbearing decisions are usually made based on current familial circumstances that relate to the composition of children already present in the family [26, 28].

Our study has unique strengths and limitations. First, with baseline and endline data and retrospective birth histories, we are able to use event history analytical techniques to estimate parity progression among fecund women conditional on sex composition. Second, most studies of son preference focus on rural areas or on nationally representative samples. Our study however, uses data from a large sample of women from six cities in Uttar Pradesh. Using data from urban areas allows us to analyze reproductive behavior and son preference in areas where the spread of modernization and changing values over family size and composition ideals is already under way. To elaborate, urban areas have value systems that are markedly different from their rural counterparts which have been found to be influential in women’s decisions regarding their family [29]. While this modern outlook might reduce parental preference for sons [11, 29], others show that son preference is intensified in urban areas [2]. Our analysis confirms the latter view: son preference is strong in this urban context which makes having only daughters less desirable.

Our study also has its limitations. First, our sample is restricted to women who were not sterilized, menopausal or pregnant at baseline. We use this restricted sample in order to use valid fertility intentions in our analysis of the relationship between sex composition and fertility. However, we acknowledge that we lose information about the sex composition and son preference of women who were already sterilized at baseline. Second, this study could also be strengthened if we had dynamic measures of fertility intentions. As is well-known, intentions measured at a particular time point could be unreliable as fertility intentions are fluid [30]. Lastly, our study does not attempt to demonstrate the influence of sex-selective abortions on women’s reproductive behavior. While this is a pervasive practice in much of northern India, we are unable to examine its role on women’s fertility in our analysis.

## Conclusions

Our study highlights that son preference is a pervasive phenomenon in India that is not restricted to rural areas. While enabling women to meet their reproductive preferences should be an intrinsic social welfare goal, policies need to be in place that help parents appreciate the value of a daughter. Many such laws are already in place in India, such as the Preconception and Prenatal Diagnostic Techniques Act of 1994 that prohibits the use of sex-selection technologies to abort a female fetus. Programs that work explicitly to enhance the value of girls can also alleviate the perceived burden of daughters to parents. Over time as these types of programs are rolled out, there will be a need to undertake similar analyses to the ones presented here to determine whether policies and programs lead to changes in son-preference in urban (and rural) India.

## Abbreviations

ICRW: International Center for Research on Women
MLE Project: Measurement, Learning & Evaluation Project
TFR: Total fertility rate
UHI: Urban Health Initiative
URHI: Urban Reproductive Health Initiative

## Footnotes

1 Ground truthing is a process whereby after using satellite imagery or government lists to identify densely populated areas/slums, the field teams visited the cities and confirmed that the identified areas were indeed slums and identified the exact boundaries of identified slums.
